# Neural responses in human superior temporal cortex support coding of voice representations

**DOI:** 10.1371/journal.pbio.3001675

**Published:** 2022-07-28

**Authors:** Kyle Rupp, Jasmine L. Hect, Madison Remick, Avniel Ghuman, Bharath Chandrasekaran, Lori L. Holt, Taylor J. Abel

**Affiliations:** 1 Department of Neurological Surgery, University of Pittsburgh, Pittsburgh, Pennsylvania, United States of America; 2 Department of Communication Science and Disorders, University of Pittsburgh, Pittsburgh, Pennsylvania, United States of America; 3 Department of Psychology, Carnegie Mellon University, Pittsburgh, Pennsylvania, United States of America; 4 Department of Bioengineering, University of Pittsburgh, Pittsburgh, Pennsylvania, United States of America; New York University, UNITED STATES

## Abstract

The ability to recognize abstract features of voice during auditory perception is an intricate feat of human audition. For the listener, this occurs in near-automatic fashion to seamlessly extract complex cues from a highly variable auditory signal. Voice perception depends on specialized regions of auditory cortex, including superior temporal gyrus (STG) and superior temporal sulcus (STS). However, the nature of voice encoding at the cortical level remains poorly understood. We leverage intracerebral recordings across human auditory cortex during presentation of voice and nonvoice acoustic stimuli to examine voice encoding at the cortical level in 8 patient-participants undergoing epilepsy surgery evaluation. We show that voice selectivity increases along the auditory hierarchy from supratemporal plane (STP) to the STG and STS. Results show accurate decoding of vocalizations from human auditory cortical activity even in the complete absence of linguistic content. These findings show an early, less-selective temporal window of neural activity in the STG and STS followed by a sustained, strongly voice-selective window. Encoding models demonstrate divergence in the encoding of acoustic features along the auditory hierarchy, wherein STG/STS responses are best explained by voice category and acoustics, as opposed to acoustic features of voice stimuli alone. This is in contrast to neural activity recorded from STP, in which responses were accounted for by acoustic features. These findings support a model of voice perception that engages categorical encoding mechanisms within STG and STS to facilitate feature extraction.

## Abstract 2

Voice perception occurs via specialized networks in higher order auditory cortex, but how voice features are encoded remains a central unanswered question. Using human intracerebral recordings of auditory cortex, this study provides evidence for categorical encoding of voice.

## Introduction

Vocalizations are a crucial social signal and a fundamental driver of human and animal behavior. Humans and other animals can easily distinguish con-specific vocalizations from other complex sounds in their acoustic environment [1,2] and can deduce demographic, emotional, and behavioral intentions from voice [3]. These voice recognition abilities begin to develop prenatally [4], precede development of linguistic abilities [5], and are formed through the processing of acoustic and paralinguistic aspects of voice [6]. However, the neural organization of auditory cortex underlying human voice perception remains a central unanswered question.

Neuroimaging studies have identified regions of auditory cortex theorized to mediate voice processing. These regions include superior temporal sulcus (STS) and superior temporal gyrus (STG), collectively referred to as “temporal voice areas” (TVAs) [7–12] and demonstrate a robust BOLD response when listening to voice stimuli compared to nonvoice stimuli. Recent neuroimaging work suggests that TVAs exist in multiple primate species [1,2,12,13]. Furthermore, this region seems to categorize conspecific vocalizations apart from other primate vocalizations, a pattern that is preserved across species [2].

These regions exhibit robust connectivity with auditory regions in the supratemporal plane (STP), including Heschl’s gyrus (HG), and higher order association cortices implicated in voice perception and voice identity recognition[14–18]. Bilateral STS exhibits voice selective responses, although some studies suggest hemispheric asymmetry in STS anatomic structure and function [10,19]. Current understanding of regional voice selectivitity and temporal dynamics of these responses in auditory cortex is driven primarily by neuroimaging studies. Therefore, studies employing methods on physiologic timescales are needed to further examine the contribution of STS and STG to voice perception.

Whether activity in putative voice-selective areas of STG and STS is actually demonstrating selectivity for voice, and not the acoustic or linguistic properties of speech, remains under debate [12,20,21]. A speech-driven model of voice coding is supported by some neuroimaging studies [8,17,20,22,23]. Extant behavioral work also suggests that voice perception may rely heavily on linguistic content [24]. However, other studies have shown voice selectivity persists when controlling for the unique acoustic properties of voice stimuli [12]. At a broader level, it remains unknown to what extent processing of complex auditory stimuli, such as speech, music, or naturally occurring environmental sounds, rely on shared or unique neural mechanisms [18,20,25–27] and how these mechanisms are organized across the auditory cortical hierarchy [28–32]. Together, support for TVAs suggests that populations of neurons in STG/STS exhibit specialization for the rich information carried by vocalizations and substantiates the hypothesis that cortical representations of vocalizations represent categorical encoding (voice versus nonvoice) beyond the contribution of encoding of vocal acoustics. Specific features driving neural encoding of voice and the timing and organization of this coding will also be advanced by approaches with greater temporal resolution.

To understand the cortical representation of voice at physiologic timescales, we measured local neural activity directly from the STS, STG, and surrounding auditory cortex in patient participants undergoing clinical intracerebral recordings as part of epilepsy surgery [33]. To date, evidence for voice coding in human auditory cortex has largely come from functional magnetic resonance imaging (fMRI) studies. The low temporal resolution of fMRI limits interpretation of temporal dynamics of these responses, given physiologic delay and low-pass filtered nature of BOLD responses compared to peak spike frequency [22,34]. Here, we leverage intracerebral recordings that uniquely allow direct electrophysiological measurements across the auditory hierarchy including from sulcal banks, such as the STS and HG. We combined this recording technique with decoding models to measure how distinguishable, across channels, the neural responses are between voice and nonvoice sounds, as well as encoding models to estimate the stimulus features driving neural responses. Additionally, we performed single-channel analyses to examine voice separability, or the degree to which a channel responds more strongly to voice than nonvoice, and voice category preference strength, or the extent to which a channel responds exclusively to voice stimuli. We test the hypotheses that vocalizations are represented categorically in STG and STS. Here, we provide data in support of voice category-level encoding in neural recordings within STG/STS and describe the temporal dynamics of these responses across temporal voice sensitive areas.

## Results

**Fig 1:**
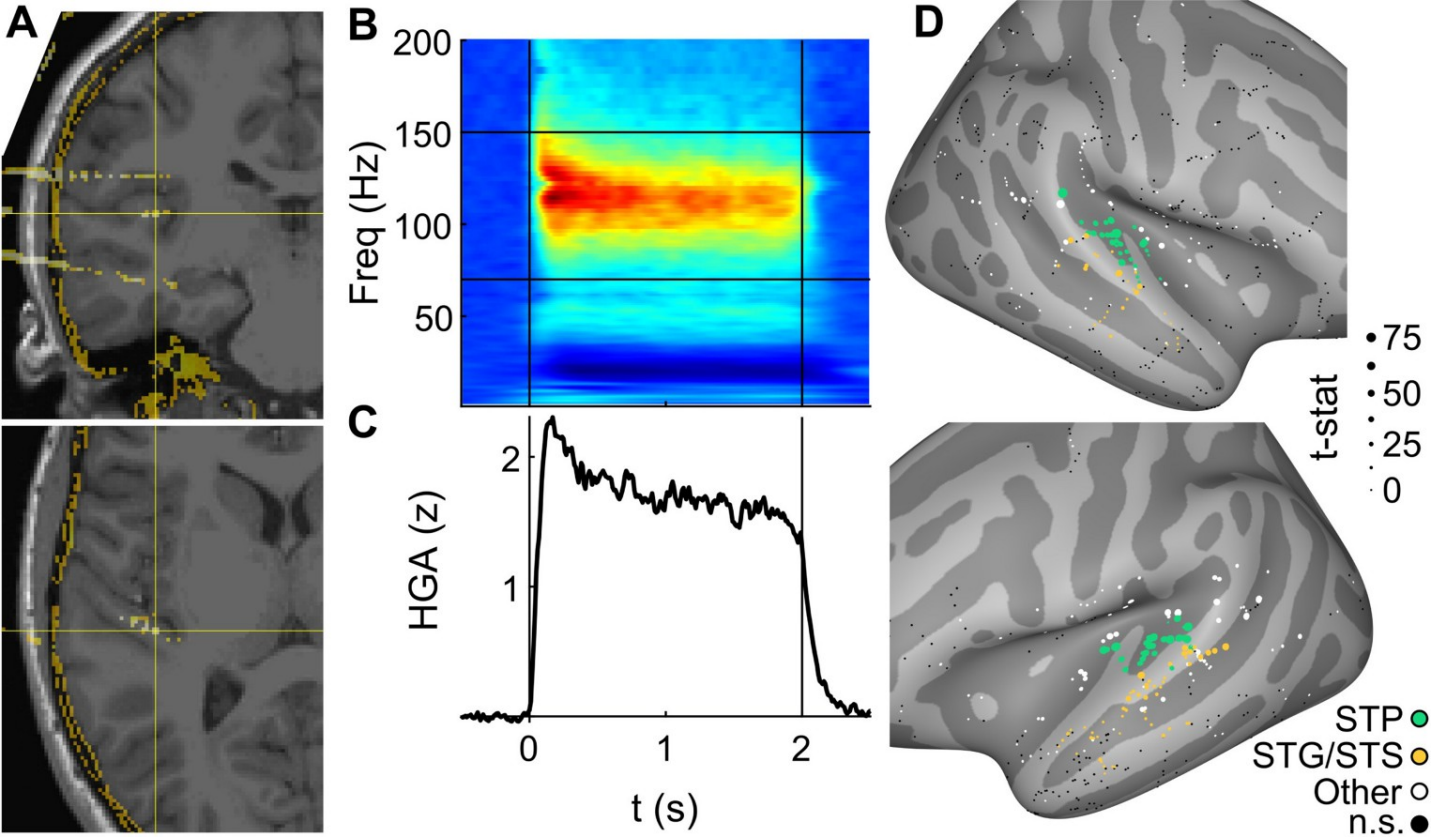
Auditory-evoked HGA. **(A)** Example channel in left HG, patient P7, shown in coronal (upper panel) and axial (lower) slices. **(B)** Auditory-evoked spectral response averaged across all NatS stimuli in channel from (A). Vertical lines represent stimulus on- and offset, with horizontal lines demarcating frequency boundaries for broadband HGA at 70 and 150 Hz. **(C)** Mean HGA in the same channel. **(D)** Auditory responsiveness, quantified as the 2-sample t-value between mean HGA in 500 ms pre- and poststimulus onset windows. Small black dots represent channels with no auditory response, i.e., t-values that failed to reach significance (*p* < 0.05, FDR corrected). Associated data are located on Zenodo in the Fig 1B and 1C folder (doi: 10.5281/zenodo.6544488). FDR, false discovery rate; HG, Heschl’s gyrus; HGA, high-gamma activity; NatS, Natural Sounds; STG, superior temporal gyrus; STP, supratemporal plane; STS, superior temporal sulcus.

We recorded neural data from 8 patient-participants (ages 9 to 18 years) undergoing intracerebral recordings as part of routine epilepsy surgery evaluation. Recording sites in each participant included STP, STG, and STS, including HG (Fig 1A). Participants performed an auditory 1-back task of natural sounds stimuli adapted from Norman-Haignere and colleagues [20] (*n* = 8; Natural Sounds, NatS). A subset of 3 participants additionally performed a 1-back task using stimuli adapted from Belin and colleagues [7] (*n* = 3; Voice Localizer, VL). Each of these stimulus sets include vocal and nonvocal sounds that can be used to assess vocal selectivity similar to previous studies [7–34]. To measure local neuronal responses to auditory stimuli, broadband high-gamma activity (HGA) [35–37], or HGA (70 to 150 Hz), was extracted (Fig 1B and 1C). We focused on HGA because it is an effective index of local neuronal processing, as it is highly correlated with local neuronal spiking activity [38]. While other frequency bands or aspects of the field potential (e.g., phase) have often been implicated in auditory cortical processing, they are outside the scope of this work. Each channel’s auditory responsiveness was assessed using a 2-sample *t* test that compared mean HGA between a poststimulus onset window (0 to 500 ms) and a silent baseline (−600 to −100 ms relative to onset). Channels that exhibited a significant auditory response (*p* < 0.05, false discovery rate [FDR] corrected) were included in subsequent analyses. This resulted in between 28 and 72 auditory-responsive channels per patient, for a total of 399 channels with NatS recordings and 174 channels with VL recordings.

### Decoding voice from nonvoice acoustic stimuli

We sought to establish the magnitude and temporal dynamics of ensemble neural response differences between vocal and nonvocal sounds, which we refer to as voice decoding. For each patient, HGA from all auditory-responsive channels was used to decode between voice and nonvocal sounds using 2 types of models. Windowed models were built using mean HGA within a sliding window (width of 100 ms, overlapping and sliding by 50 ms), with individual models built at each time window. Full models refer to those that used all windows simultaneously.

**Fig 2:**
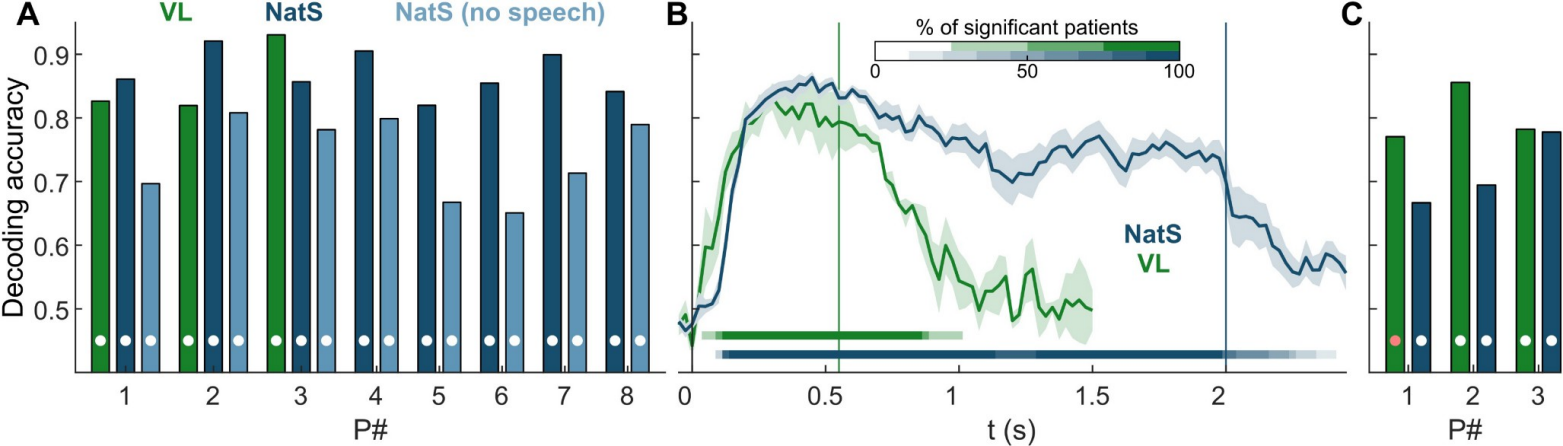
Decoding accuracy results. **(A)** Full model (i.e., all channels and time windows) decoding accuracy of vocal versus nonvocal for each patient. Dark and light blue bars correspond to NatS results with speech stimuli included or excluded, respectively (e.g., light blue is nonspeech human vocalizations versus nonvocal auditory stimuli). White dots represent statistical significance (*p* < 0.01, Bonferroni corrected, permutation tests). **(B)** Sliding window results. Vertical lines represent stimulus offset for the 2 tasks, with horizontal lines showing fraction of patients with statistically significant decoding in that window (*p* < 0.001, FDR corrected, cluster-based permutation tests). **(C)** Cross-task decoding accuracy, with color indicating the training set (white: *p* < 0.01, red: *p* < 0.05, Bonferroni corrected, permutation tests). Associated data are located on Zenodo in the Fig 2 folder (doi: 10.5281/zenodo.6544488). FDR, false discovery rate; NatS, Natural Sounds; VL, Voice Localizer.

For full models (Fig 2A), classification accuracy reached significance in each participant at *p* < 0.01 (permutation tests, Bonferroni corrected), with accuracy ranging from 82% to 93% for the VL stimulus set and 82% to 92% for NatS stimuli (see “Acoustic stimuli” section). To address whether these results were driven by encoding of linguistic information (i.e., speech) rather than voice, the full model decoding analysis was performed again with NatS data, excluding all speech stimuli (i.e., native and foreign speech, lyrical music), so that the vocal category contained only nonspeech vocal sounds such as laughter, crying, and coughing. Accuracy ranged from 65% to 80% and remained statistically significant for all patients (*p* < 0.01, Bonferroni corrected) (light blue bars, Fig 2A). It is worth noting that NatS full models may have benefitted from the longer stimuli relative to VL, which resulted in more temporal windows and therefore more input features to the decoding model.

Time courses of decoding accuracy are shown in Fig 2B, averaged across all patients for both NatS and VL. Across patients and stimulus sets, significant decoding emerged as early as 50 ms (range of 50 to 150 ms), with decoding accuracy falling below chance between 25 ms before to 500 ms after stimulus offset. Decoding accuracy trajectories were significant for the duration of the stimulus length for both NatS and VL, demonstrating that once voice decoding emerges, it persists for the duration of the sound.

Next, we examined the generalizability of our findings across different stimulus sets for the 3 participants that performed both VL and NatS tasks. Most notably, cross-task decoding shows that models trained on data from one stimulus set were able to decode vocal category membership (vocal–nonvocal, V–NV) on data from the other stimulus set (Fig 2C). Similar HGA response properties to voice were observed between tasks, despite completely distinct stimulus sets. Additionally, the sliding window accuracy profiles between VL and NatS tasks were highly correlated within patient during the VL stimulus window (550 ms; R = 0.80, 0.81, and 0.95 respectively, Fig 2B). While VL stimuli were substantially shorter than NatS, the rapid onset of significant decoding for both stimulus sets and close correspondence in their temporal profiles (Fig 2B) suggests that this difference does not lead to meaningful differences in neural responses during the first 550 ms.

### Distribution of voice-sensitive channels

**Fig 3:**
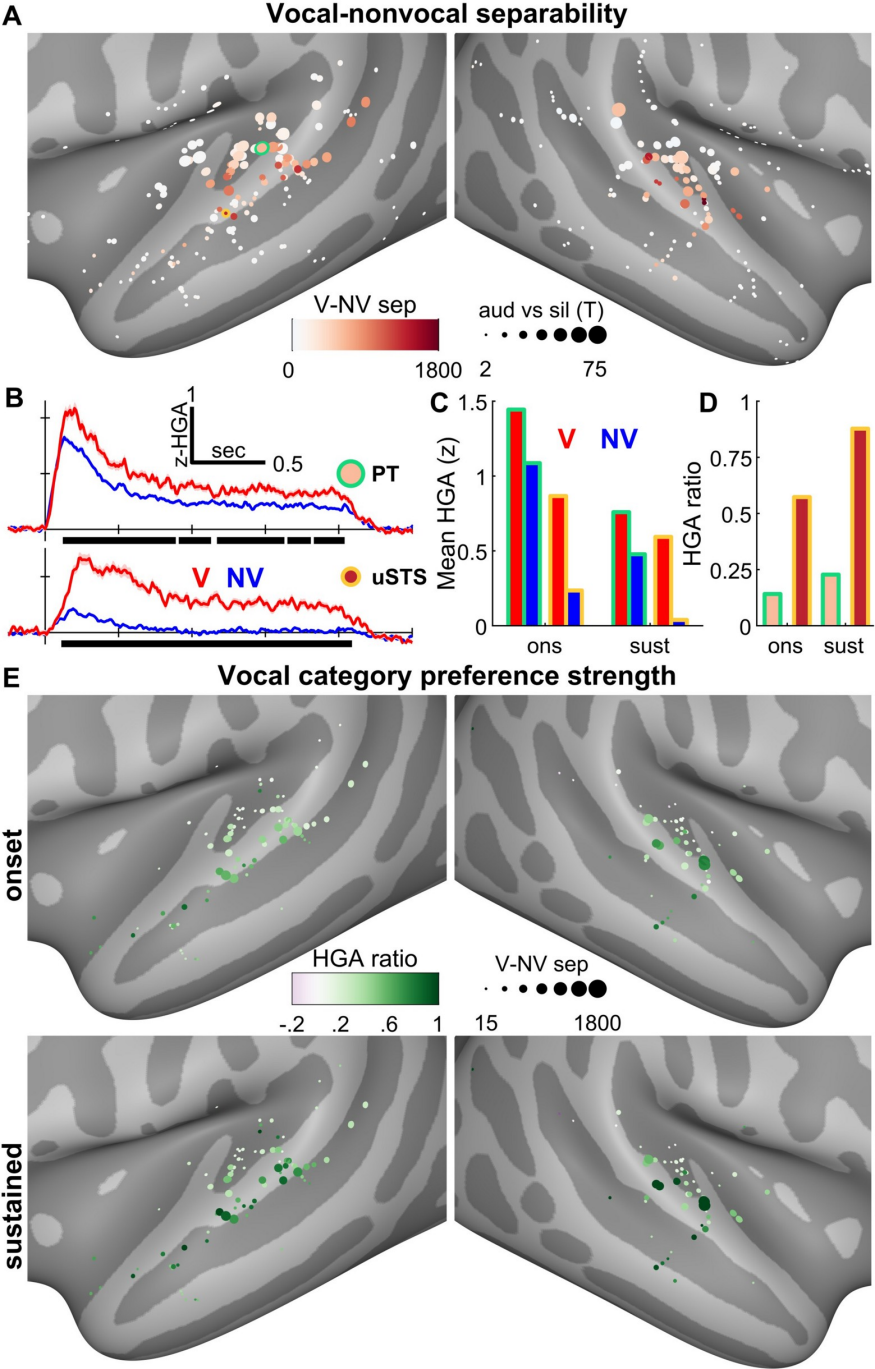
Single channel results. **(A)** HGA separability between vocal and nonvocal NatS stimuli, across all patients. Channel sizes are proportional to t-statistics comparing auditory response magnitude between 500 ms pre- and poststimulus onset windows, same as Fig 1D. **(B)** HGA for 2 example channels located in PT (upper panel) and uSTS (lower panel). Black bars show clusters of significantly different timepoints; V–NV separability (panels A, E) is the sum of all clusters for a given channel. Note that while both channels achieve V–NV separability throughout the duration of the stimulus, the magnitude of the nonvocal response differs between the 2 channels, with the NV response of the uSTS channel returning near baseline after the initial onset window. In contrast, the V response remains elevated in both onset and sustained windows, for both the PT and uSTS channels. **(C)** Mean HGA averaged across 2 different windows: onset (0 to 500 ms) and sustained (500 to 2,000 ms). **(D)** The HGA ratio is calculated as the difference between vocal and nonvocal responses, relative to their sum. This metric, spanning from −1 to 1, describes a channel’s vocal category preference strength: a value near 1 (or −1) represents a channel that responds only to vocal (or nonvocal) stimuli, while a value of 0 represents equal HGA responses to both stimulus categories. **(E)** All channels with V–NV separability exhibit onset responses to both stimulus categories: in this early window, HGA ratios reveal that STG and STS (compared to STP) shows a slightly diminished response to nonvocal relative to vocal stimuli. During the sustained window, a strong preference for vocal stimuli emerges in STG and STS, while nonvocal responses return near silent baseline. Associated data are located on Zenodo in the Fig 3B–3D folder (doi: 10.5281/zenodo.6544488). HGA, high-gamma activity; NatS, Natural Sounds; PT, planum temporale; STG, superior temporal gyrus; STP, supratemporal plane; STS, superior temporal sulcus; uSTS, upper STS; V–NV, vocal–nonvocal.

Next, to test the hypothesis that vocal separability, or the extent to which a channel shows greater HGA responses to voice than nonvoice sounds, is driven by activity in STG and STS, we compared HGA between individual cortical recording sites in STP (comprised of HG and planum temporale, PT) and STG/STS. Across all channels, STG/STS had smaller overall auditory HGA responses (*p* < 10^−5^, rank-sum test) relative to STP. Although we had hypothesized that V–NV separability would be greater in STG/STS than STP, we found that there was no statistically significant difference between these 2 regions of interest (ROIs; both auditory responsiveness and V–NV separability shown in Fig 3A). We suspected that the latter finding may have been driven by a significant difference in the proportion of channels showing significant V–NV separability (87% STP versus 66% STG/STS; *p* = 0.004, Fisher exact test). However, even when excluding nonsignificant channels, V–NV separability was not significantly different between STG/STS and STP.

V–NV separability describes how distinguishable vocal responses are from nonvocal responses but does not characterize the extent to which a channel responds exclusively to only vocal (and not nonvocal) natural sounds; we refer to the latter characteristic as the voice category preference strength. To illustrate the difference, consider the 2 channels in Fig 3B. Both channels exhibit V–NV separability because there is a relative difference between voice and nonvoice responses. However, a major qualitative difference exists between the 2 channels: In the PT channel (upper plot), responses to both stimulus categories are elevated above baseline throughout the stimulus duration; in contrast, the upper STS (uSTS) channel (lower plot) shows elevated responses to only the vocal category, particularly after an initial onset window in which all natural sounds elicit some response. To characterize the category preference strength (i.e., the extent to which a channel responds exclusively to vocal sounds), we quantified the HGA ratio—a metric of the normalized neural response—as the difference of mean V and NV responses relative to their sum. Responses that are more exclusively confined to only V stimuli exhibit HGA ratios closer to 1, while ratios close to 0 represent much weaker category preference.

As alluded to in the previous paragraph, responses exhibited 2 windows of distinct activity, consisting of an onset (0 to 500 ms) and a sustained response (500 to 2,000 ms). In order to characterize the voice category preference strength in each of these windows separately, mean HGA was calculated for each category (V and NV) and temporal window (Fig 3C, using the same channels as Fig 3B). These values were then used to calculate HGA ratios, shown in Fig 3D. Notice that the PT channel displays a weak category preference (HGA ratio < 0.25) throughout the stimulus duration, while the uSTS channel transitions from a moderate to a strong category preference between the onset and sustained window.

Fig 3E shows HGA ratio results across all separable channels (those with nonzero V–NV separability), revealing that the trend from Fig 3B–3D generalizes across channels. Specifically, across the auditory hierarchy, V–NV separability in the onset window is broadly driven by a weak category preference, strengthening to a stronger preference in the sustained window, i.e., HGA ratios are greater in the sustained relative to the onset window (*p* = 1.6 × 10^−5^, sign-rank test). Furthermore, STG/STS channels displayed a stronger vocal category preference relative to STP channels in both windows (*p* < 10^−5^ for both onset and sustained, rank-sum tests). In summary, HGA responses are more exclusive to vocal sounds in STG/STS relative to STP, as well as in the sustained relative to the onset window.

Last, the onset of V–NV separability was also estimated in STP and STG/STS. This onset is highly sensitive to the response strength, i.e., channels with poor signal-to-noise might show later separability onsets due to noise contamination. Therefore, we calculated the median onset time for only the 50% most separable channels in a given ROI. This resulted in median onsets of HGA V–NV separability of 130 ms in STP and 150 ms in STG/STS.

### Voice feature encoding demonstrates category-level representation of voice in the STS

Finally, we investigated whether categorical voice responses could be explained by lower-level acoustic processing. To this end, we used the audio processing software OpenSMILE to extract acoustic features of varying complexity [39,40]. Specifically, we used the functionals feature set, which produces statistical summaries (e.g., mean, standard deviation, and peak rate) of acoustic features (e.g., loudness, formants, mel-frequency cepstral coefficients, jitter, and shimmer) for each stimulus. An additional binary feature was included to indicate vocal category membership. Encoding models were built to predict onset and sustained mean HGA (as in Fig 3C) for each channel showing V–NV HGA separability in auditory cortex using this feature space. Full encoding models used the full feature space, while nested encoding models were built without the categorical voice feature.

Two metrics were derived from this analysis that, when taken together, provide insight into a channel’s encoding properties. First, the percent of variance explained (R^2^) by the full model describes how well the input features explain the response magnitudes for a given channel. Second, the likelihood ratio test compares the nested to the full model and provides an estimate of the added value conferred by the introduction of the vocal category membership feature. Under the null hypothesis that both models fit the data equally well, this ratio is χ^2^ distributed.

**Fig 4:**
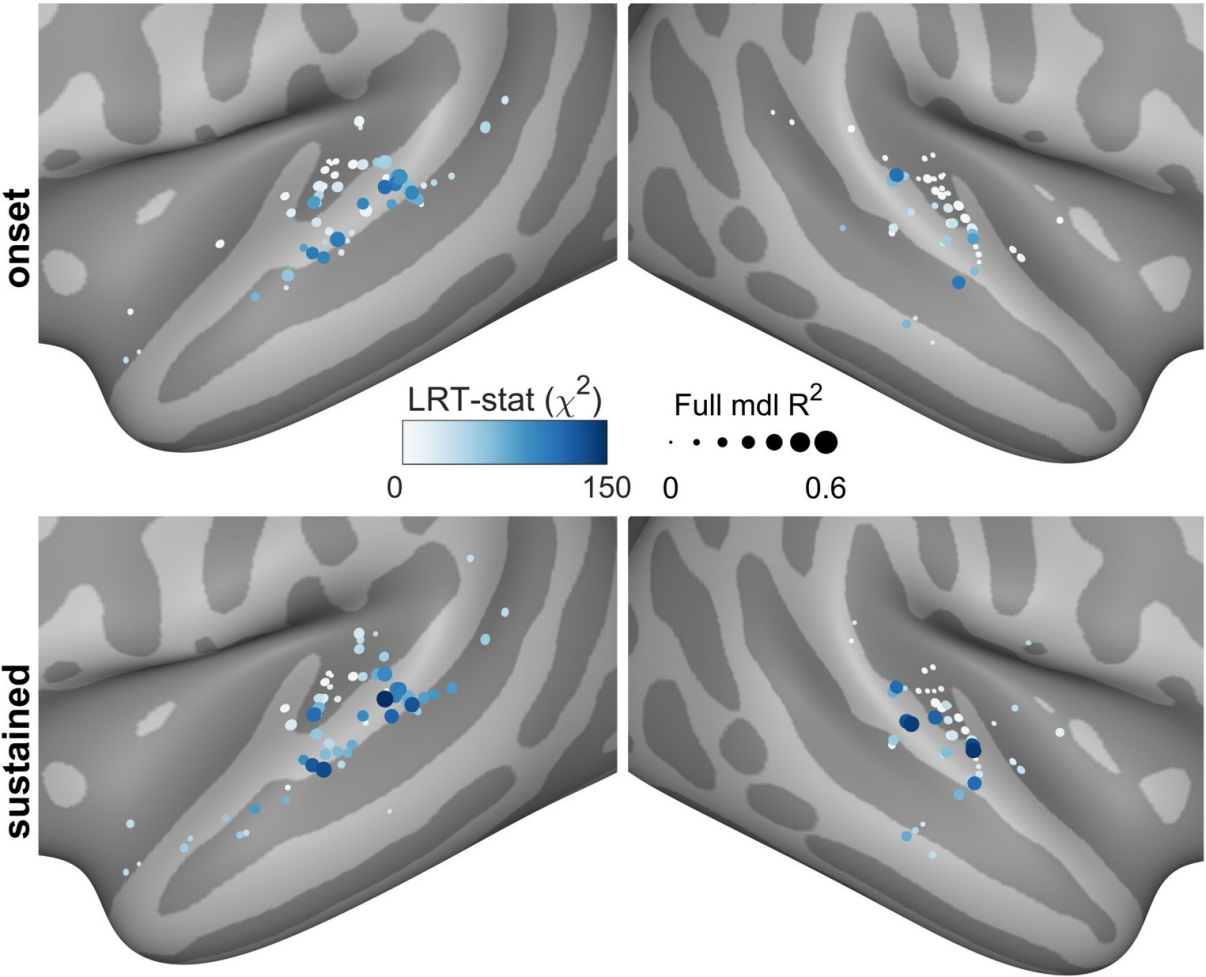
Encoding model results. Linear regression encoding models suggest that STP is primarily driven by acoustic features, while STG and STS responses are much more influenced by category-like information. Model inputs consisted of both low- and high-level acoustic features such as loudness, MFCCs, spectral flux, and relative formant ratios. Full models also included a binary feature indicating vocal category membership. Likelihood ratio test statistics compare this full model to a nested, acoustic-only model and thus describe the improvement conferred by V–NV class information. Well-fit channels in STP are modeled best by acoustic features throughout both the onset and sustained windows. Meanwhile, STG and STS channels also perform well and benefit from the addition of category-level information, with a slight skew toward the later sustained window. STG, superior temporal gyrus; STP, supratemporal plane; STS, superior temporal sulcus; V–NV, vocal–nonvocal.

Among full encoding models with significant R^2^ values (*p* < 0.05, Bonferroni corrected, permutation tests), 2 qualitatively different types of responses emerged in the STP and STG/STS. The first group, clustered in STP, represents auditory feature encoding and is characterized by a combination of large R^2^ and low χ^2^ values (Fig 4). These channels are well explained by encoding models in both the onset and sustained windows but show minimal improvement in model performance when a vocal category feature is added. The second group of channels, clustered in lateral STG and STS, demonstrates categorical encoding properties by showing substantial model improvement (large χ^2^) with the addition of categorical voice information (as well as large R^2^ values). ROI analysis confirms that χ^2^ values were significantly larger in STG/STS compared to STP in both the onset (*p* < 10^−5^) and sustained windows (*p* < 10^−5^, rank-sum tests). R^2^ values from full (acoustic + category) encoding models were not significantly different between regions in either window. In contrast, acoustic-only R^2^ values were significantly larger in STP compared to STG/STS in both the onset (*p* < 10^−5^) and sustained windows (*p* < 10^−5^, rank-sum tests).

## Discussion

The human TVAs have long been associated with vocal category preference, but the exact computations underlying this phenomenon remain debated [20,24,27,41]. One hypothesis is that, similar to face processing, TVAs perform a voice detection gating function in which incoming auditory stimuli are categorized as vocal or nonvocal prior to higher level feature extraction [18,42–44]. If such a model were correct, one would expect category-level encoding of vocal stimuli in TVAs. Our results demonstrate cortical regions in the STG and STS, the putative sites of TVAs, have strong vocal category preference across 2 distinct voice localizer tasks, NatS and VL. Notably, the data show that voice selectivity strengthens along the auditory cortical hierarchy from STP to STG/STS in a temporally dynamic fashion, with an initial less specific onset response followed by a sustained response with pronounced voice category preference in STG/STS [45]. Auditory sEEG experimental data presented here demonstrate category selectivity to voice even in participants as young as 9, suggesting functional specialization of the STG/STS arises prior to adulthood. Importantly, our results demonstrate that separability between HGA reponse to voice and nonvoice acoustic stimuli in the STG/STS is driven most robustly by voice category, rather than lower-level acoustic features. In contrast, voice-sensitive sites in the STP were driven primarily by acoustic features rather than voice category.

Whether voice selectivity in human auditory cortex is driven by low-level acoustic features or whether neural response selectivity actually reflects more abstract representation of voice category remains an open question [12,20,24]; some have suggested that voice specialization actually reflects specialization for speech [20,22–24]. First, our results show that while speech information plays a role in vocal neural coding, vocal decoding can occur even in the absence of speech. Second, our encoding model results demonstrate that in the STG/STS voice category plays a far more influential role than low-level acoustic features. Thus, our results support a model in which there is a gradient of voice category selectivity across the auditory hierarchy, with lower-level acoustic features playing the most important role in STP, and strong voice category selectivity emerging in bilateral STG/STS. Last, we found no significant hemispheric differences (left versus right STP plus STG/STS) in any metrics explored here, including V–NV separability, HGA ratio, and encoding model metrics (R^2^ and χ^2^). These findings are in line with other work showing no left-right asymmetries in vocal category encoding [8]; however, this does not preclude the possibility of hemispheric specialization for higher level voice representations such as speaker identity.

This study also sheds light on critical temporal dynamics of voice processing across the auditory system. Previous high density EEG work reported an N200 signal distinguishing vocal from nonvocal stimuli with an onset at 164 ms [46]. Meanwhile, a subsequent MEG study showed a dissociation in the neural activity of vocal and nonvocal activity starting at 150 ms [47]. These studies both inferred a similar localization of this effect: The MEG study showed maximal dissociation around bilateral STS, while the HD-EEG study also proposed a similar anatomical locus. Norman-Haignere and colleagues examined temporal integration in STP and STG/STS across diverse timescales and found that channels with “short-integration” windows (<200 ms) show selectivity for spectrotemporal modulation, while “long-integration” channels (>200 ms) show prominent category selectivity [32]. In agreement with these findings, we observed an onset of V–NV separability around 150 ms in STG/STS channels. Interestingly, our decoding results revealed a slightly earlier onset across auditory cortex, starting between 50 and 150 ms. These results may be due to decoding models exhibiting higher sensitivity to early weak separability, given the inclusion of multiple channels simultaneously.

While we found that separability between vocal and nonvocal responses exists throughout auditory cortex, several response characteristics suggest that voice selectivity is localized in STG/STS. Vocal category preference strength and categorical voice encoding are both stronger in this region, particularly during the sustained window following onset responses. In support of this, Bodin and colleagues provides further evidence that TVAs encode ecologically salient vocalization categories by showing selectivity of anterior STG/STS to conspecific vocalizations over vocalizations from other primate species (human, macaque, and marmoset) [2]; for review, see Bodin and Belin [13]. In contrast, STP sites that display V–NV separability show a weak category preference strength, possibly related to acoustic feature encoding rather than true category specificity. This explanation is supported by the finding that responses in this region encode acoustic features more strongly than categorical features. Additionally, our results support the idea of dynamic selectivity in the STS (i.e., there are 2 distinct phases of selectivity), whereby vocal category preference strength evolves from weak during the onset window to strong during the sustained response.

The NatS stimulus set was not designed as a voice localizer and thus possesses a lower proportion of nonspeech vocal stimuli. To ensure this stimulus set functioned similarly as a purpose-built TVA localizer (like VL), we performed a cross-decoding analysis between the natural sounds and voice localizer paradigms, which show that responses to vocal versus nonvocal sounds are similar across these separate stimulus sets (Fig 2C). This stands in contrast to functional neuroimaging work that used the NatS stimulus set to show that temporal voice regions may not exist [20]. Notably, Norman-Haignere and colleagues [27] recently published on intracranial recordings to the same NatS stimulus set and showed voice selectivity not driven by speech. They suggested that the disparity between these results and previous fMRI work could be related to the relatively reduced granularity of fMRI approaches compared to sEEG. A recent fMRI study using artificially generated sounds demonstrated that temporal voice regions may encode vocal perceptual quality, i.e., the extent to which a sound is voice like [21]. Since the environmental sounds stimuli do not sufficiently sample across this perceptual continuum, the current data are unable to shed light on this possibility directly. However, a weak (STP) versus strong (STG/STS) category preference strength could reflect encoding of acoustic and perceptual features respectively.

We demonstrate dynamic category-driven encoding of voice in the human STG/STS. Further, with the spatiotemporal resolution of intracerebral recordings, our results demonstrate a gradient of selectivity across auditory processing regions with distinct temporal dynamics underlying different aspects of voice processing. Taken together, our findings support a voice gating mechanism of voice coding by temporal voice regions.

## Materials and methods

### Participants and electrode implantation

sEEG recordings of the STS, STG, and STP (including HG) were performed in 8 neurosurgical patients with drug-resistant epilepsy as part of clinical evaluation for epilepsy surgery. See Table 1 for patient-participant characteristics. Written informed consent and assent (for patients >14 years old) was obtained from all participants. The research protocol was approved by the University of Pittsburgh Institutional Review Board (STUDY20030060).

**Table 1:** Participant epilepsy and behavioral characteristics.

	Summary	P1	P2	P3	P4	P5	P6	P7	P8
Age (years)	15 (3)	16	16	9	18	13	15	15	14
Female, *n* (%)	2 (25)	Male	Male	Male	Male	Female	Male	Female	Male
Right handed, *n* (%)	7 (87)	Left	Right	Right	Right	Right	Right	Right	Right
WASI-II score	85 (16)	76*	62*	67	95	94	81	102	104
Hemisphere language	5 (63)	Left	Left	Left	Right	Bilateral	Left	Right	Left
Epilepsy onset (years)	8 (5)	1	0	8	12	3	10	13	13
Seizure locus		Right parietal lobe	Right temporal lobe	Frontal lobe	Left frontotemporal, HG	Right Amygdala	Mesial temporal lobe	Mesial temporal lobe	Left temporal gyrus
Epilepsy etiology		Unknown	Unknown	Autoimmune encephalitis	Autoimmune encephalitis	Mesial temporal sclerosis	Heterotopias and FCD	FCD	Pilocytic astrocytoma
Electrode contacts (auditory responsive/all)		72/256	70/226	32/120	37/127	28/104	55/117	58/117	47/52
NatS performance (% correct)	85 (16)	100	99	55.6	74.7	73.7	93.9	96	90.9
NatS median RT (ms)	1,153 (346)	929	1,291	1,064	1,145	1,912	818	1,174	887
VL performance (% correct)	88 (15)	100	71.4	91.1	-	-	-	-	-
VL median RT (ms)	837 (136)	682	898	932	-	-	-	-	-

Summary statistics reported as mean (SD) unless otherwise specified.

*WISC-V.

FCD, focal cortical dysplasia; HG, Heschl’s gyrus; NatS, Natural Sounds; RT, reaction time; VL, Voice Localizer.

All patients underwent preoperative neuropsychological evaluation. sEEG electrode implantation was performed as previously described [33]. Briefly, Dixi Medical Microdeep electrodes were used, with a diameter of 0.8 mm, contact length of 2 mm, and center-to-center spacing of 3.5 mm. Electrodes contained between 8 and 18 contacts each, which we refer to as channels. For 6 of 8 patients, there was incomplete sampling of implanted channels due to clinical hardware constraints (recording maximum of 128 channels, well below the number typically implanted). Clinical staff generally chose to record every other channel on a given electrode shaft, although they occasionally strayed from this heuristic for clinical reasons.

### Data collection

Patients performed an auditory 1-back task using short clips of natural environmental sounds (see next section for more details). Audio was presented binaurally via Etymotic ER-3C earphones, with volume adjusted to a comfortable level for each patient separately before the start of the experiment(s). Inter-stimulus intervals were randomized uniformly between 1 and 2 seconds. Patients were instructed to indicate 1-back stimulus repeats using a button box (Response Time Box, v6). Repeats occurred on about 16% of all trials. Each stimulus was presented a minimum of 2 (VL) or 3 (NatS) times, although the 1-back task design resulted in some stimuli with more presentations (up to 4 for VL and 5 for NatS).

Neural data were recorded at 1 kHz using the Ripple Grapevine Nomad system (model R02000) with a real-time notch filter applied at 60, 120, and 180 Hz. For patients P3 to P8, the audio signal was split using a commercial splitter with separate volume controls; for P1 and P2, the audio was fed into a distribution amplifier (Rolls, model DA134). In both cases, the split audio signal was presented to patients and simultaneously recorded synchronously with neural data by the Ripple amplifier.

### Acoustic stimuli

Two different types of stimuli were used in separate experiments, which we refer to as VL and NatS. VL stimuli were modified versions of the stimuli used in the Belin voice localizer [7]. These original stimuli were designed for fMRI experiments and consisted of 8-second clips, with each clip containing a series of either vocal (e.g., speech, laughter, and coughing) or nonvocal (e.g., mechanical, music, and animal vocalizations) sounds only. These stimuli were adapted to capitalize on the temporal resolution afforded by intracranial research: PRAAT silence-based segmentation was used to extract and save individual sounds from each clip [48]. Sounds with duration shorter than 550 ms were discarded; all other sounds were shortened to this duration, linear-ramped on and off by 50 ms, and rms-normalized. This procedure generated 80 nonvocal and 72 vocal sounds; to ensure balanced classes, only the first 72 nonvocal sounds were selected.

NatS stimuli were the same as those originally used by Norman-Haignere and colleagues [20]. Each of the 165 sounds are 2 seconds in duration and belong to 1 of 11 categories, defined in the original study, which we grouped into superordinate categories of vocal and nonvocal sounds. Vocal categories consisted of English and foreign speech, human vocalizations, and lyrical music. Similar to VL, nonvocal sounds were more varied and included categories such as mechanical sounds, nonlyrical music, and animal vocalizations.

Importantly, the NatS stimulus set contained human nonspeech vocal sounds that might not activate voice-selective regions of cortex. Specifically, crowd-generated cheering and laughter may be categorically different from vocal sounds generated by individuals. Furthermore, following the heuristic outlined by Belin and colleagues [7], we excluded sounds without vocal fold vibrations, namely breathing and whistling. Based on these 2 considerations, we reclassified 4 NatS stimuli from the vocal to the nonvocal category.

### Anatomy

For each patient, cortical surfaces were reconstructed from a preoperative MRI using Freesurfer [49]. Using the MATLAB third-party package Brainstorm, the MRI was then co-registered with a postoperative CT scan, and channels were localized. MNI normalization was performed using Brainstorm’s implementation of SPM12’s nonlinear warping. This MNI deformation field was then used to warp the Julich volumetric atlas into patient space [50–52], and each channel was localized to an ROI by finding the ROI label of the closest voxel. ROI labels for each channel were visually inspected and manually corrected where appropriate.

### Data preprocessing

A common average reference (CAR) filter was used to remove noise common across channels. While bipolar montages have been shown to result in improvements across some signal metrics [53], a CAR filter was chosen given the incomplete channel sampling described earlier. Voltages were epoched by extracting a window of 1,000 ms before stimulus onset to 1,450 ms after offset in the case of VL or 1,000 ms after offset for NatS. Each channel was then normalized relative to the prestimulus period across all trials. All channels whose centroid was further than 3 mm from the closest cortical vertex (either the pial surface or the gray-white matter boundary) were excluded.

To estimate broadband HGA, epoched data were forward- and reverse-filtered using a bank of 8 bandpass Butterworth filters (sixth order), with log-spaced center frequencies (70 to 150 Hz) and bandwidths (16 to 64 Hz). The analytic signal amplitude was extracted using the Hilbert transform. Each band was then normalized relative to a common baseline across all trials; in estimating the mean and standard deviation for normalization, the earliest 100 ms of baseline were discarded due to edge effects, and the 100 ms immediately preceding stimulus onset were discarded to prevent any contamination from low-latency responses. HGA was then calculated as the mean across these 8 bands, which was down-sampled to 100 Hz and clipped to a window of 900 ms before onset to 900 ms after offset.

Auditory-responsive channels were identified using 2-sample *t* tests comparing mean HGA between a 500 ms window immediately following stimulus onset to a baseline period defined as −600 to −100 ms preonset. Only channels with *p* < 0.05 (FDR corrected) were used in subsequent analysis. For patients that completed both VL and NatS, channels were labeled auditory-responsive if they surpassed this threshold in at least one of the 2 tasks. At this point, HGA was averaged across all presentations of a stimulus, which we refer to as a stimulus response. Unless otherwise noted, stimulus responses (as opposed to single-trial responses) were used in all subsequent analysis.

### Decoding analysis

For each patient, decoding was performed via L1-regularized logistic regression using the MATLAB package *glmnet*. Input features consisted of mean HGA in 100 ms windows, sliding every 50 ms. Relative to stimulus onset, window centers spanned from −50 to 1,500 ms for VL and −50 to 2450 ms for NatS. Full models that included all channels and time windows, as well as sliding models that used single windows, were built. While the duration of VL (550 ms) and NatS stimuli (2,000 ms) differed, recent evidence suggests that this might not have resulted in a meaningful difference between NatS and VL sliding window decoding results (for the first 550 ms). Using the same NatS stimuli as this study, Norman-Haignere and colleagues demonstrated that temporal integration windows across auditory cortex showed an upper bound of 400 ms [45]; since both stimulus sets exceeded this window length, they both produced responses in which the sounds were entirely contained within each channel’s integration window. In contrast, NatS full models may have benefitted from the longer stimuli, which resulted in more temporal windows and therefore more input features to the decoding model.

In addition to regularization, cross validation (5-fold) was used to prevent overfitting and explore generalizability. Within each cross-validation fold, 20% of data was held out in a testing set; the remaining 80% was further split into a 72% training and 8% validation set using a 10-fold inner-loop cross validation scheme. Before the inner-loop cross validation, input features in the training+validation and testing sets were z-scored relative to the training+validation set. Additionally, each observation was weighted by the inverse of its class prevalence to prevent models from biasing toward the most numerous class; these weights were calculated using the training + validation set. This step was especially important for NatS, due to a large class imbalance (37 vocal, 128 nonvocal stimuli). Last, the inner-loop cross validation was used to test 20 different regularization parameters; the parameter was selected based on the model that minimized the mean deviance across inner folds. Balanced decoding accuracies were reported, in which the within-class accuracy for vocal and nonvocal stimuli were calculated separately and then averaged. Nonspeech vocal decoding (Fig 2A, light blue bars) was performed using single-trial (as opposed to stimulus) responses, due to the scarcity of NSV exemplars in the NatS stimulus set. For cross-task decoding, input features were limited to the shorter stimulus duration of VL, i.e., only windows within the first 550 ms. A single model was built on all data in one task and then tested on all data in the other task.

Statistical significance was assessed for sliding window decoding via a permutation-based clustering approach [54]. Briefly, V–NV labels were shuffled, and sliding window decoding was performed 1,000 times. At each window, this generated separate accuracy null distributions from which critical values were drawn, identified as the upper 95th percentile. For each permutation, values that exceeded their window’s critical threshold were saved, and temporally adjacent values were summed to create a cluster mass. The max cluster mass for each permutation was stored, generating a null distribution of 1,000 cluster masses (if a permutation contained no windows that exceeded threshold, the max cluster mass was set to 0). Finally, the same procedure was applied to the true (unshuffled) sliding window accuracies, and each resultant cluster mass was assigned a *p*-value equal to the proportion of null cluster masses that exceeded it.

### Single-channel analysis

Single channel V versus NV separability was estimated using a similar approach. However, rather than using decoding accuracy, time-varying 2-sample t-statistics were calculated on single-trial responses, and 10,000 permutations were used. V–NV separability (Fig 3A) was quantified as the sum cluster mass, i.e., the sum of masses for all significant (*p* < 0.001) clusters for a given channel. The importance of using this metric (as opposed to the max cluster mass) can be appreciated in the upper panel of Fig 3B: Summing across this channel’s 5 separate clusters gives a more accurate description of the overall separability.

Across V–NV separable channels, HGA response profiles appeared to share consistent morphological characteristics, namely onset and sustained responses of varying magnitudes. The longer stimulus durations in the NatS stimulus set provide a better estimate of sustained response properties and is thus the focus of this analysis. We first averaged HGA across an onset window (initial 500 ms following stimulus onset) and a sustained window (remainder of the stimulus length, 500 to 2,000 ms) and then calculated the mean HGA within V and NV stimuli. This window length is supported by a recent study that found that lateral STG exhibited integration windows that spanned up to about 500 ms poststimulus onset (integration window length plus neural response delay) [45]. While these integration windows varied substantially across auditory cortex, we opted for a conservative window length of 500 ms to separate onset responses, where the integration window might still contain the prestimulus baseline, from sustained responses, where the integration window fully overlaps with the stimulus.

To investigate response characteristics to V versus NV stimuli, we then calculated the HGA ratio, defined as the difference in mean HGA between these stimulus categories, normalized to the sum of these responses. Normalization helps account for a potential signal-to-noise confound: If a given channel’s overall response is scaled, the V–NV difference will be amplified as well.


$$r_{HGA}=\frac{HGA_{v}-HGA_{nv}}{HGA_{v}+HGA_{nv}}$$

Assuming positive values for both HGA_v_ and HGA_nv_, this measure ranges between −1 and 1, with a value near 1 indicating a strong preference for vocal stimuli and a value near −1 indicating a strong preference for nonvocal stimuli. Among auditory-responsive channels that also demonstrated V–NV separability, a small fraction of them (7%) exhibited negative mean HGAs across NV stimuli, representing a NV-associated decrease in the HGA response relative to baseline. To constrain the HGA ratio between −1 and 1, these values were set to 0 before the calculation.

### Encoding models

By averaging across stimulus categories, the acoustic variability between individual stimuli has thus far been ignored. One possibility is that channels exhibiting a strong preference for vocal sounds are actually encoding lower-level acoustic properties that are inherently different between vocal and nonvocal sounds. To explore this possibility, encoding models were used to predict stimulus responses, i.e., mean HGA in both onset and sustained windows, for each channel that exhibited V–NV separability. Our approach closely mirrored the encoding model methods reported by Staib and Fruholz [21].

The OpenSMILE acoustic processing package, implemented in python, was used to extract the “functionals” set of 88 acoustic features for each NatS stimulus [39,40]. These features consist of statistical summaries (e.g., mean, standard deviation, and percentiles) of acoustic features of varying complexity (e.g., loudness, mel-frequency cepstral coefficients, spectral flux, and formant values). This feature space contained a high degree of collinearity between features; therefore, we used principal component analysis to reduce its dimensionality. The first *n* principal components that captured 99.99% of the variance in the original feature space were kept. Last, a categorical feature was added indicating vocal category membership. One stimulus (chopping food) was removed due to outliers in its acoustic features.

Linear regression encoding models were then built in 1 of 2 ways, corresponding to 2 relevant measures of interest. First, the overall model fit was calculated as the out-of-sample R^2^ value using leave-one-out cross-validation. Statistical significance of R^2^ values was assessed using Bonferroni-corrected *p*-values generated from permutation tests with 10,000 permutations, in which rows of the feature matrix were shuffled before model building.

Second, the likelihood ratio test statistic was calculated between the full model and a nested version that excluded the vocal category feature. This statistic estimates the likelihood that the vocal category feature provides additional information beyond the acoustic features and is χ^2^ distributed under the null hypothesis. Log likelihoods for both full and nested models were attained from models trained on full stimulus sets.

## Abbreviations

CAR: common average reference
FDR: false discovery rate
fMRI: functional magnetic resonance imaging
HG: Heschl’s gyrus
HGA: high-gamma activity
NatS: Natural Sounds
PT: planum temporale
ROI: region of interest
STG: superior temporal gyrus
STP: supratemporal plane
STS: superior temporal sulcus
TVA: temporal voice area
uSTS: upper STS
VL: Voice Localizer
V–NV: vocal–nonvocal
